# Comparative speed of kill of induced *Ixodes scapularis* infestations following treatment of dogs with endectocides containing either sarolaner (Simparica Trio™), afoxolaner (NexGard^®^ PLUS), or lotilaner (Credelio Quattro™)

**DOI:** 10.1186/s13071-026-07559-y

**Published:** 2026-07-17

**Authors:** Kathryn E. Reif, Todd M. Kollasch, Casey L. Locklear, Matthew D. Eberts, Kayla N. Braswell, Anastasia M. W. Cooper, James W. Gillespie, Brian H. Herrin, Jonas P. McAbee, Carter M. Morgan, Anna Grace Morrow, Bailey J. Sperling, Daniel J. Sweeney, William G. Ryan

**Affiliations:** 1 Department of Pathobiology, College of Veterinary Medicine, Auburn University, Auburn, AL 36849 USA; 2 Elanco Animal Health, 450 Elanco Circle, Indianapolis, IN 46221 USA; 3 Department of Biomedical and Diagnostic Sciences, College of Veterinary Medicine, University of Tennessee, Knoxville, TN 37996 USA; 4 Ryan Mitchell Associates LLC, 16 Stoneleigh Park, Westfield, NJ 07090 USA

**Keywords:** Afoxolaner, Blacklegged tick, Canine, Credelio Quattro, Efficacy, *Ixodes scapularis*, Lotilaner, NexGard PLUS, Sarolaner, Simparica Trio

## Abstract

**Background:**

*Ixodes scapularis* is a consequential tick species in North America that can transmit multiple pathogens of canine and public health significance. Acaricidal speed is critical for reducing the risk of pathogen transmission. Herein, the immediate and sustained acaricidal speed of three monthly oral endectocide products against *I. scapularis* are compared.

**Methods:**

Thirty-two dogs were randomly allocated equally among four groups and treated on day 0 with an endectocide containing lotilaner (Credelio Quattro™), afoxolaner (NexGard**®** PLUS) or sarolaner (Simparica Trio™), or left untreated. On days −2, 21, and 28, dogs were infested with 50 laboratory-reared, adult *I. scapularis*. Live tick counts were performed at 4, 8, 12, 24, and 48-h post-treatment, and after each reinfestation. Efficacy was compared using a linear mixed model of geometric mean live tick counts.

**Results:**

At 8-h post-treatment of an existing infestation, only the lotilaner group had significantly lower mean live tick counts than the untreated group. At each subsequent assessment through 48 h, all treated groups had significantly lower mean live tick counts than the untreated group. Compared to the untreated group, following the day 21 reinfestation, mean live tick counts were significantly lower in the lotilaner group at 4 through 48 h, in the sarolaner group at 4, 24, and 48 h, and in the afoxolaner group at 24 and 48 h. Compared to the untreated group, following the day 28 reinfestation, mean live tick counts were significantly lower in the lotilaner group at 8 through 48 h, in the sarolaner group at 12 through 48 h and, in the afoxolaner group at 24 and 48 h. Within group, between day 0 and day 28, significant slowing of acaricidal speed was observed for sarolaner at the 12-h timepoint and for afoxolaner at the 8, 12, and 24-h timepoints. No significant loss of acaricidal speed occurred for lotilaner between day 0 and day 28.

**Conclusions:**

In dogs, lotilaner has a faster onset of acaricidal activity against *I. scapularis* than sarolaner or afoxolaner. Lotilaner’s acaricidal speed is sustained throughout the monthly dosing interval, whereas sarolaner and afoxolaner show slower acaricidal activity at the end of the month than at the beginning.

**Graphical Abstract:**

**Figure:**
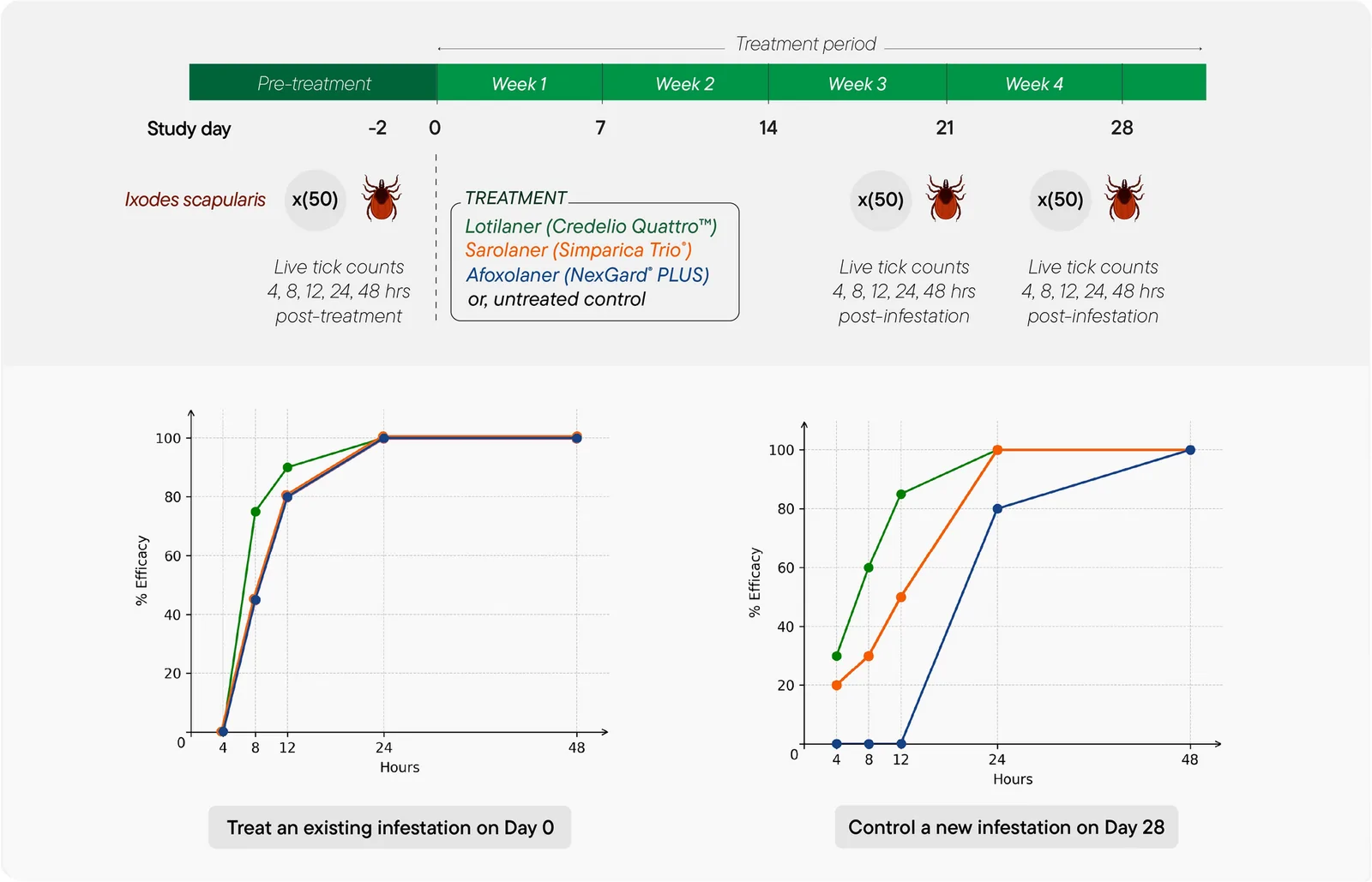

**Supplementary Information:**

The online version contains supplementary material available at https://doi.org/10.1186/s13071-026-07559-y.

## Background

Ongoing geographic range expansion of *Ixodes scapularis* Say (Acari: Ixodidae) in North America is driven by increasing forest cover, expanding white-tailed deer populations, and rising global temperatures [1–3]. As the primary vector of numerous tick‐borne pathogens, the expanding range of *I. scapularis* has been accompanied by increased transmission of tick-borne pathogens, many of which are zoonotic and of considerable importance to human and animal health [4–6]. Pathogens commonly transmitted by *I. scapularis* include agents of Lyme borreliosis (*Borrelia burgdorferi, Borrelia mayonii),* and anaplasmosis (*Anaplasma phagocytophilum*), and less commonly, agents of babesiosis (*Babesia microti*), ehrlichiosis (*Ehrlichia muris eauclairensis*), and Powassan virus disease [4, 7]. Reported human Lyme disease diagnoses increased from approximately 30,000 in 2010 to more than 89,000 in 2023, while reported human anaplasmosis cases rose from 273 to 7,280 between 2000 and 2023 [5, 6]. Coinfection of *I. scapularis* ticks with multiple pathogens is also reported to occur commonly [1, 8]. The growth in human cases of Lyme borreliosis across a broader geography parallels an increase in pathogen transmission to canines infested with *I. scapularis* [9]. Because the risk of pathogen transmission increases with the duration of tick attachment, treatments with rapid acaricidal activity are needed to protect dogs at risk of tick exposure.

Since first being marketed in 2014, isoxazoline compounds have become a common choice for veterinary-prescribed canine tick and flea control programs. Each isoxazoline was originally marketed as a single-entity product. Recently, three of these isoxazolines—afoxolaner, sarolaner, and lotilaner—were combined with moxidectin and pyrantel and registered in the USA to provide monthly control of canine heartworm disease and intestinal parasitic nematodes, while retaining ectoparasiticidal activity. The lotilaner-containing product also includes praziquantel, for cestode treatment and control. The minimum doses of afoxolaner (2.5 mg/kg) and lotilaner (20 mg/kg) in these combination products remain the same as their respective single-entity product, while the minimum sarolaner dose was reduced from 2.0 mg/kg in the single-entity product to 1.2 mg/kg in the combination product [10–12].

The acaricidal speed (i.e., how quickly an active ingredient kills ticks) of these isoxazolines throughout the month-long post-treatment period is important in reducing the risk of canine tick-borne pathogen infection. Studies have shown substantial variation in the acaricidal speed between these compounds against existing infestations, and against reinfestations that occur later during the labeled treatment period [13–18]. Comparison studies of the available monthly dosed isoxazoline products evaluating their acaricidal speed could assist veterinarians in providing evidence-based guidance on tick control recommendations to their dog-owning clients. The objective of the study reported here was to compare the acaricidal speed of three, monthly administered combination products in dogs challenged with *I. scapularis* prior to and during the labeled treatment period.

## Methods

This randomized complete block design, positive and negative controlled, investigator-masked study was conducted in accordance with Good Clinical Practice standards and the World Association for the Advancement of Veterinary Parasitology (WAAVP) guidelines for evaluating the efficacy of parasiticides for the treatment, prevention, and control of flea and tick infestations of dogs and cats [19, 20]. All study personnel involved in tick infestations, counts, and viability assessments, were masked to the treatment of each dog. Staff administering treatment, or with knowledge of any treatment, took no part in post-treatment tick counts or viability assessments. For personal protection, all staff wore protective gloves and gowns that were changed regularly to prevent inadvertent product transfer between dogs in different groups. The study was conducted after ethical approval was received from the Elanco Animal Welfare Authority and in full compliance with an approved Institutional Animal Care and Use Committee Protocol (IACUC Protocol# 2025-5686) on file with Auburn University in Auburn, AL.

### Dogs and management

Thirty-eight purpose-bred Beagle dogs, approximately 7 to 8 months old, were acclimated in the study facility for one week prior to study start. After the acclimation period, an *I. scapularis* test infestation was performed on all dogs on day −7 (with day 0 being treatment day). From the 38 dogs, 32 were selected for the study. The six dogs excluded from the study had either the lowest tick counts 48 h after the day −7 test infestation or had behavioral reasons that would have made tick counting too difficult. For inclusion, dogs had to be clinically healthy with no pre-existing conditions (e.g., injury, trauma, disease) that could affect study outcomes, be non-pregnant and non-lactating, weigh at least 2.0 kg, have demonstrated a susceptibility to tick infestation based on pre-treatment tick counts (retaining ≥ 25% of the tick infestation challenge), and be negative for *Dirofilaria immitis*, *A. phagocytophilum*, *Borrelia* spp., and *Ehrlichia* spp. based on a blood test (SNAP 4Dx Plus, IDEXX Laboratories Inc., Westbrook, ME, USA) completed during acclimation. Dogs were excluded if they had a poor hair coat indicative of a dermatological condition, a known history or behavior that could affect the study outcome, known previous exposure to ticks, treatment with an isoxazoline in the 12 months prior to starting the study or with any topically applied acaricide/insecticide within the 60 days prior to starting the study, or wearing or had removed an acaricidal/insecticidal collar within the previous 60 days.

For the entire study, dogs were housed in individual indoor cages in a thermostatically controlled environment. Visual contact was possible between dogs. Temperature and humidity were recorded daily. The dogs were provided with an approximately 12/12 h light/dark cycle. After randomization to treatment groups, physical contact between dogs in different groups was not possible. Water was provided ad libitum in bowls, and a commercial ration was provided daily for each dog, according to the manufacturer’s recommendation for adequate diet. On the morning of administration of the study treatments (day 0), food was withheld from all dogs until approximately 30 min before the scheduled time of treatment. Daily general health observations were completed on each day of acclimation and continued throughout the study duration for the 32 enrolled dogs.

### Tick infestations

Adult *Ixodes scapularis* ticks were purchased from Oklahoma State University and maintained in humidity chambers at > 90% relative humidity and room temperature until used in the study. At the time of the study, the adult ticks were 12–16 weeks post-molt. This *I. scapularis* colony was initiated in 1991 with engorged females that were field-collected in Stillwater, Oklahoma. The colony is refreshed yearly with wild engorged female *I. scapularis* collected in Oklahoma. Prior to infestations, the ticks were counted into containers of approximately 35 females and 15 males. Female and male ticks had been maintained separately until infestation. During acclimation and on day -2, day 21, and day 28, each dog was infested with approximately 50 ticks (35 female and 15 male)*.* For each infestation, a masked assessor exhaled slowly over the top of the opened container to stimulate tick activity. Non-sedated dogs were manually restrained, and ticks were deposited onto the dorsal midline, starting between the shoulder blades. To facilitate tick attachment, each dog was confined in an appropriately sized travel crate for approximately 4 h immediately following the infestation procedure and then returned to its cage.

### Randomization and treatment

The 32 enrolled dogs, 15 males and 17 females, aged 7 to 8 months, all intact, were ranked and placed into eight blocks of four based on descending live tick counts. Using Microsoft Excel, a random number was assigned to each dog within a given block. The highest random number in each block was assigned to Group 1, the second-highest to Group 2, and so on through Group 4. Dogs randomized to Group 1 remained untreated but were handled in the same manner as those receiving any treatment (i.e., offered food and removed from pens to simulate treatment activities) to provide a time reference for post-treatment activities (i.e., tick infestation, tick counting). Groups 2, 3, and 4 were treated per label on day 0, based on the each dog’s respective body weight obtained from a calibrated scale on day −2. Group 2 dogs were treated with the combination product containing sarolaner (Simparica Trio, Zoetis), Group 3 dogs were treated with the combination containing afoxolaner (NexGard PLUS, Boehringer Ingelheim) and Group 4 dogs received the combination containing lotilaner (Credelio Quattro, Elanco Animal Health). Each dog consumed at least half of its daily calories within 30 min pre-treatment. According to group allocation, each dog received a single tablet of their group’s respective product, appropriate to meet at least the labeled minimum target dosage. Treatment administrators wore gloves, changed between each dog, and gowns, changed between each group. Each dog was observed for any adverse events (e.g., vomiting) for 5 min post-treatment, and at 1, 2, and 4 h post-treatment (all ± 30 min).

### Tick counts

Tick counts were conducted by teams of two or three people, using gentle manual restraint of non-sedated dogs. Counting teams were rotated to ensure objective tick assessment at each tick count time point. The procedure involved running gloved fingers or a flea comb against the lay of the hair to expose any ticks. Examinations began with the dog’s head, including inside the ears, then along the neck and back, each flank, abdomen, inguinal areas, under the tail, chest including axillae, front legs and paws, and hind legs and paws, including examination between digits on each paw. Each dog was examined for at least 5 min (average evaluation time for each dog was approximately 20 min). Any tick observed to have any leg movement, however slight, was classified as live. A tick was classified as dead if no movement was displayed, including leg motion when stimulated (breathing on the tick, probing with fingers or forceps). Ticks were also recorded as being attached or unattached, and male or female. Tick counts were completed at 4, 8, 12, and 24 h post-treatment and after each subsequent infestation. Following the final assessment after each infestation, all ticks were counted and removed with forceps; 48 ± 2 h post-treatment and 48 ± 2 h post-reinfestation.

### Statistical analysis

Descriptive statistics (geometric mean, arithmetic mean, standard deviation, minimum, median, and maximum values) were calculated for each treatment group and timepoint. Efficacy of each product was calculated using the formula:

$${\text{Efficacy }}\left( \% \right) \, = { 1}00 \, \times \, \left( {Mc - Mt} \right)/Mc,$$

where *Mc* is the mean number of live ticks on dogs in the untreated control group and *Mt* is the mean number of live ticks on dogs in a treated group. The primary efficacy objective was based on geometric mean live tick counts, considered the most appropriate measure of central tendency [19].

Tick counts were analyzed separately for each infestation and timepoint using linear mixed models. Each model had a fixed effect of treatment group and a random effect of block. Multiple comparisons between treatments were adjusted for with Tukey’s test at each time point separately. Error term variance was modeled as heterogeneous with respect to treatment group if there was evidence of heterogeneous variance. For efficacy assessments based on geometric means, counts of live ticks were subjected to ln(tick counts + 1) transformation before statistical analysis. Untransformed standard errors were calculated using the delta method on model based standard errors. As a sensitivity analysis, efficacy assessments were also calculated based on arithmetic means with counts of live ticks modeled directly without any transformation. A significance level of 0.05 was used. All analyses were performed using SAS 9.4 (Cary, NC).

To determine if there was a significant decline in acaricidal speed with time after treatment, the acaricidal speed on day 0 was compared with the acaricidal speed on day 28 at the 4, 8, 12, 24, and 48-h timepoints using the natural log(tick counts + 1). Prior to the analysis, the counts were normalized by subtracting the transformed mean untreated control value at each day and timepoint for each dog. The difference between day 0 and 28 was calculated for each dog and timepoint. Within each product-treated group, differences were assessed by a paired t-test or the Wilcoxon signed rank test, depending on distribution of the data. Normality of the differences was assessed by examining histograms and QQ-plots.

## Results

Assessment of pre-study blood samples from study dogs found no evidence of prior exposure to *Borrelia* spp., *Anaplasma* spp., *Ehrlichia* spp., or *D. immitis*. Pre-treatment body weights between groups ranged from 6.5 to 9.2 kg (median 7.5 kg) in the control group, 6.8 to 8.6 kg (median 7.3) in the sarolaner group, 6.9 to 9.9 kg (median 7.9 kg) in the afoxolaner group, and 6.9 to 9.3 kg (median 8.1 kg) in the lotilaner group. For the sarolaner group, the administered sarolaner dosage ranged from 1.2 to 1.7 mg/kg (median 1.5 mg/kg, minimum label dosage 1.2 mg/kg); for the afoxolaner group the administered afoxolaner dosage ranged from 2.5 to 4.8 mg/kg (median 2.7 mg/kg, minimum label dosage 2.5 mg/kg); for the lotilaner group, the administered lotilaner dosage ranged from 24.3 to 32.7 mg/kg (median 27.9 mg/kg, minimum label dosage 20 mg/kg). All products were administered per product label, with food provided to all dogs within 30 min before treatment. There were no events of treatment-related vomiting, regurgitation, or lost doses. No dog required treatment re-administration. Across all study assessments, live tick counts in the untreated control group ranged from 14 to 43, mean counts from 23.0 to 36.0, thereby meeting the required minimum count (≥ 25% of each infestation of 50 ticks—i.e., 13 ticks) to allow conclusions concerning the efficacy of each product at every study assessment.

### Speed of tick kill to treat an existing *Ixodes scapularis* infestation

At 4 h post-treatment, at least 14 live ticks were counted on each study dog (Table 1). At 8 h post-treatment, only in the lotilaner group were geometric mean live tick counts significantly lower than in the control group (Table 2; Supplementary table S1). At each subsequent assessment through 48 h post-treatment, mean live tick counts in all groups were significantly lower than in the control group, with no significant between-group differences in mean live tick counts (Table 2).

**Table 1:** Mean live tick counts of dogs with existing infestations of *Ixodes scapularis*, placed on day −2, left untreated or treated on day 0 with either sarolaner, afoxolaner or lotilaner

Hours post-treatment		4	8	12	24	48
Untreated	Number of dogs tick free	0	0	0	0	0
	Range	14–33	25–39	17–43	28–41	24–39
	Median	29	33	32.5	36	37
	Arithmetic mean (SE)	26.5 (2.1)	32.1 (2.7)	30.8 (2.1)	35.5 (0.8)	35.6 (0.9)
	Geometric mean (SE)	25.6 (2.1)	31.9 (6.2)	29.3 (7.7)	35.3 (4.2)	35.3 (2.2)
Sarolaner (Simparica Trio)	Number of dogs tick free	0	0	0	6	8
	Range	20–30	5–28	1–6	0–1	0–0
	Median	23.0	20.5	2.5	0.0	0.0
	Arithmetic mean (SE)	24.1 (2.1)	18.4 (2.7)	2.7 (2.1)	0.2 (0.8)	0.0 (0.9)
	Geometric mean (SE)	23.8 (1.9)	16.0 (3.1)	2.5 (0.6)	0.2 (0.02)	0.0 (0.0)
Afoxolaner (NexGard Plus)	Number of dogs tick free	0	0	2	7	7
	Range	15–38	7–29	0–15	0–1	0–1
	Median	29.5	20.0	4.0	0.0	0.0
	Arithmetic mean (SE)	28.5 (2.1)	17.8 (2.7)	5.3 (2.1)	0.1 (0.8)	0.2 (0.9)
	Geometric mean (SE)	27.7 (2.3)	16.3 (3.1)	3.1 (0.8)	0.1 (0.01)	0.2 (0.01)
Lotilaner (Credelio Quattro)	Number of dogs tick free	0	0	1	6	8
	Range	20–38	1–30	0–10	0–3	0–0
	Median	28.5	8.5	4.5	0.0	0.0
	Arithmetic mean (SE)	28.1 (2.1)	10.6 (2.7)	4.5 (2.1)	0.6 (0.8)	0.0 (0.9)
	Geometric mean (SE)	27.7 (2.3)	8.1 (1.6)	3.1 (0.8)	0.4 (0.05)	0.0 (0.0)

SE standard error

8 dogs per group; Infestations with 50 *I. scapularis* ticks

**Table 2:** Summary statistics with degrees of freedom (DOF) for sarolaner (Simparica Trio), afoxolaner (NexGard PLUS) and lotilaner (Credelio Quattro) groups at hours following day 0 treatment of an existing *Ixodes scapularis* infestation

Hours	Treatment group	GM (± SE)	Compared with control		Compared with sarolaner	Compared with afoxolaner
			Test statistic (DOF) *P*-value	Efficacy (%)	Test statistic (DOF) *P*-value	Test statistic (DOF) *P*-value
4	Untreated control	25.6 (2.1)				
	Sarolaner	23.8 (1.9)	0.58(21); 0.938	6.7		
	Afoxolaner	27.7 (2.3)	− 0.68(21); 0.902	0.0	− 1.26(21); 0.597	
	Lotilaner	27.7 (2.3)	− 0.68(21); 0.904	0.0	− 1.26(21); 0.600	0.01(21);1.000
8	Untreated control	31.9 (6.2)				
	Sarolaner	16.0 (3.1)	2.44(21); 0.099	49.6		
	Afoxolaner	16.3 (3.1)	2.39(21); 0.110	48.8	− 0.06(21); 1.000	
	Lotilaner	8.1 (1.6)	4.77(21); < 0.001	74.5	2.32(21);0.125	2.38(21); 0.112
12	Untreated control	29.3 (7.7)				
	Sarolaner	2.5 (0.6)	5.86(21); < 0.001	91.6		
	Afoxolaner	3.1 (0.8)	5.41(21); < 0.001	89.5	− 0.45(21); 0.969	
	Lotilaner	3.1 (0.8)	5.36(21); < 0.001	89.3	− 0.49(21); 0.960	− 0.05(21); 1.000
24	Untreated control	35.3 (4.2)				
	Sarolaner	0.2 (0.02)	21.1(21); < 0.001	99.5		
	Afoxolaner	0.1 (0.01)	21.7(21); < 0.001	99.7	0.54(21); 0.949	
	Lotilaner	0.4 (0.05)	20.1(21); < 0.001	98.8	− 1.07(21); 0.710	− 1.61(21); 0.396
48	Untreated control	35.3 (2.2)				
	Sarolaner	0.0 (0.00)	40.3(21); < 0.001	100		
	Afoxolaner	0.2 (0.01)	38.4(21); < 0.001	99.5	− 1.95(21); 0.240	
	Lotilaner	0.0 (0.0)	40.3(21); < 0.001	100	0.00(21); 1.000	1.95(21); 0.240

Infestations with 50 *I. scapularis* per dog; 8 dogs in each group; SE standard error

If mean tick counts in a product-treated group were the same as or greater than in the untreated control group, efficacy is stated as 0.0%

### Speed of tick kill to control an *Ixodes scapularis* challenge 21 days post‑treatment

At 4 h post-reinfestation on day 21, at least 13 live ticks were counted on each study dog (Table 3). At this timepoint, mean live tick counts in the sarolaner and lotilaner groups were significantly lower than in the control group (Table 4). Mean live tick counts from the lotilaner group were then significantly lower than control group counts at each subsequent assessment through the final assessment at 48 h post-infestation. Sarolaner group mean live tick counts were significantly different from the control group counts at 24 and 48 h post-infestation but not at 8 or 12 h. In the afoxolaner group, mean live tick counts that were significantly lower than control group counts were observed at 24 h and 48 h post-reinfestation on day 21.

**Table 3:** Mean live tick counts of dogs left untreated or treated with sarolaner, afoxolaner or lotilaner on day 0 and infested with 50 *Ixodes scapularis* on day 21

Hours post-infestation		4	8	12	24	48
Untreated control	Number of dogs tick free	0	0	0	0	0
	Range	26–37	17–36	16–36	23–32	29–43
	Median	30.5	28.0	21.5	26.5	37.5
	Arithmetic mean (SE)	30.6 (2.2)	27.8 (2.0)	23.8 (2.2)	27.3 (2.0)	36.4 (1.0)
	Geometric mean (SE)	30.4 (2.5)	26.9 (2.6)	23.0 (4.9)	27.1 (5.7)	36.0 (1.9)
Sarolaner (Simparica Trio)	Number of dogs tick free	0	0	0	4	8
	Range	13–32	13–29	2–25	0–3	0–0
	Median	24.0	17.5	14.5	0.5	0.0
	Arithmetic mean (SE)	23.5 (2.2)	19.6 (2.0)	14.3 (2.2)	1.1 (2.0)	0.0 (1.0)
	Geometric mean (SE)	22.7 (1.8)	18.8 (1.8)	12.0 (2.6)	0.8 (0.2)	0.0 (0.0)
Afoxolaner (NexGard Plus)	Number of dogs tick free	0	0	0	0	7
	Range	22–44	23–40	15–31	1–35	0–1
	Median	31.5	30.0	23.5	6.5	0.0
	Arithmetic mean (SE)	31.5 (2.2)	30.3 (2.0)	24.3 (2.2)	9.4 (2.0)	0.1 (1.0)
	Geometric mean (SE)	30.7 (2.5)	29.9 (2.9)	23.7 (5.1)	6.0 (1.2)	0.1 (0.0)
Lotilaner (Credelio Quattro)	Number of dogs tick free	0	0	1	6	8
	Range	15–32	7–18	0–16	0–1	0–1
	Median	20	11.5	3.5	0.0	0.0
	Arithmetic mean (SE)	22.0 (2.2)	11.8 (2.0)	5.0 (2.2)	0.1 (2.0)	0.0 (1.0)
	Geometric mean (SE)	21.4 (1.7)	11.2 (1.1)	3.2 (0.7)	0.1 (0.02)	0.0 (0.0)

SE standard error

8 dogs per group; infestations with 50 *I. scapularis* ticks

**Table 4:** Summary statistics with degrees of freedom (DOF) for sarolaner (Simparica Trio), afoxolaner (NexGard PLUS) and lotilaner (Credelio Quattro) groups at hours following infestations on Day 21 with 50 *Ixodes scapularis* ticks for each dog

Hours	Treatment group	GM (± SE)	Compared with control		Compared with sarolaner	Compared with afoxolaner
			Test statistic (DOF) *P*-value	Efficacy (%)	Test statistic (DOF) *P*-value	Test statistic (DOF) *P*-value
4	Untreated control	30.4 (2.5)				
	Sarolaner	22.7 (1.8)	3.08(21); 0.027	25.3		
	Afoxolaner	30.7 (2.5)	− 0.11(21); 0.999	0.0	− 3.19(21); 0.021	
	Lotilaner	21.4 (1.7)	3.67(21); 0.007	29.4	0.60(21); 0.932	3.79(21); 0.006
8	Untreated control	26.9 (2.6)				
	Sarolaner	18.8 (1.8)	2.75(21); 0.054	30.2		
	Afoxolaner	29.9 (2.9)	− 0.81(21); 0.849	0.0	− 3.56(21); 0.009	
	Lotilaner	11.2 (1.1)	6.67(21); < 0.001	58.6	3.92(21); 0.004	7.48(21); < 0.001
12	Untreated control	23.0 (4.9)				
	Sarolaner	12.0 (2.6)	2.02(21); 0.213	47.7		
	Afoxolaner	23.7 (5.1)	− 0.08(21); 1.000	0.0	− 2.10(21); 0.186	
	Lotilaner	3.2 (0.7)	5.75(21); < 0.001	58.6	3.73(21); 0.006	5.83(21); < 0.001
24	Untreated control	27.1 (5.7)				
	Sarolaner	0.8 (0.2)	9.39(21); < 0.001	97.2		
	Afoxolaner	6.0 (1.2)	4.73(21); < 0.001	77.9	− 4.66(21); < 0.001	
	Lotilaner	0.1 (0.02)	11.0(21); < 0.001	99.7	1.64(21); 0.378	6.30(21); < 0.001
48	Untreated control	36.0 (1.9)				
	Sarolaner	0.0 (0.00)	51.57(21); < 0.001	100		
	Afoxolaner	0.1 (0.00)	50.3(21); < 0.001	99.7	− 1.24(21); 0.611	
	Lotilaner	0.0 (0.00)	51.57(21); < 0.001	100	0.00(21); 1.000	1.24(21);0.611

Infestations with 50 *I. scapularis* per dog; 8 dogs in each group; SE standard error

If mean tick counts in a product-treated group were the same as or greater than in the untreated control group, efficacy is stated as 0.0%

For between-group comparisons, mean live tick counts in the lotilaner group were significantly lower than sarolaner group counts at 8 and 12 h post-infestation, and significantly lower than afoxolaner group counts at 4, 8, 12, and 24 h post-infestation (Table 4; Supplementary table S2). Mean live tick counts in the sarolaner group were significantly lower than afoxolaner group counts at 4, 8 and 24 h post-infestation. At no timepoint were mean live tick counts in the afoxolaner group significantly lower than those of the other treated groups.

### Speed of tick kill to control an *Ixodes scapularis* challenge 28 days post‑treatment

At 4 h post-reinfestation on day 28, at least 18 live ticks were counted on each study dog (Table 5). Mean live tick counts in the lotilaner group were significantly lower than control group counts at 8, 12, 24, and 48 h post-infestation (Table 6; Supplementary table S3). In the sarolaner group, mean live tick counts were significantly lower than control group counts at 12, 24, and 48 h post-reinfestation. In the afoxolaner group, mean live tick counts were significantly lower than control group counts at 24 and 48 h post-reinfestation.

**Table 5:** Mean live tick counts of dogs left untreated or treated with sarolaner, afoxolaner or lotilaner on day 0 and infested with *Ixodes scapularis* on day 28

Hours post-infestation		4	8	12	24	48
Untreated	Number of dogs tick free	0	0	0	0	0
	Range	22–43	14–38	18–38	14–34	18–38
	Median	28.0	22.5	24.5	28.0	31.5
	Arithmetic mean (SE)	30.5 (2.4)	24.0 (2.2)	25.5 (1.7)	26.3 (1.6)	29.9 (1.2)
	Geometric mean (SE)	29.9 (2.2)	23.1 (2.6)	25.0 (4.5)	25.5 (5.8)	29.3 (3.7)
Sarolaner (Simparica Trio)	Number of dogs tick free	0	0	0	8	8
	Range	24–39	16–31	5–20	0–0	0–0
	Median	29.0	21.0	11.0	0.0	0.0
	Arithmetic mean (SE)	30.3 (2.4)	21.6 (2.2)	11.4 (1.7)	0.0 (1.6)	0.0 (1.2)
	Geometric mean (SE)	29.8 (2.2)	21.0 (2.4)	10.5 (1.9)	0.0 (0.0)	0.0 (0.0)
Afoxolaner (NexGard Plus)	Number of dogs tick free	0	0	0	3	7
	Range	23–44	18–31	14–28	0–18	0–6
	Median	28.0	24.0	25.0	4.5	0.0
	Arithmetic mean (SE)	31.8 (2.4)	24.6 (2.2)	22.8 (1.7)	5.9 (1.6)	0.8 (1.2)
	Geometric mean (SE)	30.8 (2.3)	24.1 (2.8)	22.3 (4.0)	2.9 (0.7)	0.3 (0.04)
Lotilaner (Credelio Quattro)	Number of dogs tick free	0	0	1	7	8
	Range	18–32	4–20	0–10	0–1	0–0
	Median	27.0	12.5	5.0	0.0	0.0
	Arithmetic mean (SE)	26.1 (2.4)	13.0 (2.2)	4.6 (1.7)	0.1 (1.6)	0.0 (1.2)
	Geometric mean (SE)	25.7 (1.9)	11.8 (1.4)	3.3 (0.6)	0.1 (0.0)	0.0 (0.0)

SE standard error

8 dogs per group; infestations with 50 *I. scapularis* ticks

**Table 6:** Summary statistics with degrees of freedom (DOF) for sarolaner (Simparica Trio), afoxolaner (NexGard Plus) and lotilaner (Credelio Quattro) groups at hours following infestations on day 28 with 50 *Ixodes scapularis* ticks for each dog

Hours	Treatment group	GM (± SE)	Compared with control		Compared with sarolaner	Compared with afoxolaner
			Test statistic (DOF) *P*-value	Efficacy (%)	Test statistic (DOF) *P*-value	Test statistic (DOF) *P*-value
4	Untreated control	29.9 (2.2)				
	Sarolaner	29.8 (2.2)	0.01(21); 1.000	0.1		
	Afoxolaner	30.8 (2.3)	− 0.29(21);0.991	0.0	− 0.30(21); 0.991	
	Lotilaner	25.7 (1.9)	1.38(21); 0.524	14.1	1.37(21); 0.529	1.67(21); 0.364
8	Untreated control	23.1 (2.6)				
	Sarolaner	21.0 (2.4)	0.54(21); 0.948	8.8		
	Afoxolaner	24.1 (2.8)	− 0.26(21); 0.993	0.0	− 0.81(21); 0.851	
	Lotilaner	11.8 (1.4)	3.87(21); 0.004	48.7	3.33(21); 0.015	4.14(21); 0.002
12	Untreated control	25.0 (4.5)				
	Sarolaner	10.5 (1.9)	3.54(21); 0.010	58.0		
	Afoxolaner	22.3 (4.0)	− 0.26(21); 0.993	10.9	− 3.06(21); 0.028	
	Lotilaner	3.3 (0.6)	3.87(21); 0.004	86.8	4.27(21); 0.002	7.32(21); < 0.001
24	Untreated control	25.5 (5.8)				
	Sarolaner	0.0 (0.0)	10.1(21); < 0.001	100		
	Afoxolaner	2.9 (0.7)	5.91(21); < 0.001	88.6	− 4.22(21); 0.002	
	Lotilaner	0.1 (0.0)	9.87(21); < 0.001	99.6	− 0.27(21); 0.993	3.95(21); 0.004
48	Untreated control	29.3 (3.7)				
	Sarolaner	0.0 (0.0)	19.2(21); < 0.001	100		
	Afoxolaner	0.3 (0.04)	17.8(21); < 0.001	99.1	− 1.37 (21); 0.533	
	Lotilaner	0.0 (0.0)	19.2(21); < 0.001	100	− 0.00(21); 1.000	1.37 (21); 0.533

Infestations with 50 *I. scapularis* per dog; 8 dogs in each group; SE standard error

If mean tick counts in a product-treated group were the same as or greater than in the untreated control group, efficacy is stated as 0.0%

For between-group comparisons, mean live tick counts in the lotilaner group were significantly lower than sarolaner group counts at 8 and 12 h post-infestation, and significantly lower than afoxolaner group counts at 8, 12, and 24 h post-infestation (Table 6). Mean sarolaner group live tick counts were significantly lower than afoxolaner group counts at 12 and 24 h post-infestation. At no timepoint were mean live tick counts in the afoxolaner group significantly lower than in either of the other treated groups.

### Within-group speed of tick kill comparison between day 0 vs day 28

Within each treatment group, the efficacy at 8, 12, 24, and 48 h post-treatment on day 0 was compared with the efficacy recorded after the final infestation on day 28 (Table 7). The comparison was made following normalization of control group live tick counts. For the sarolaner group, at day 28 the reduction in acaricidal speed approached significance at 8 h and was significantly slower at 12 h. For the afoxolaner group, relative to day 0, the acaricidal speed on day 28 was significantly slower at 8 h. For the lotilaner group, the acaricidal speed was not significantly different between day 0 and day 28 time points.

**Table 7:** Test statistic and degrees of freedom (DOF) for within-group comparison of tick count reductions, relative to untreated controls, on day 0 compared with day 28

Treatment group	Tick count Time (Hr)	Statistical test	DOF	Test statistic	*P*-value
Sarolaner (Simparica Trio)	4	Student’s *t*	7	0.79	0.454
	8	Student’s *t*	7	2.33	0.052
	12	Student’s t	7	8.34	< .001
	24	Signed Rank	7	3	0.797
	48				NC
Afoxolaner (Nexgard Plus)	4	Student’s *t*	7	− 0.54	0.607
	8	Student’s *t*	7	3.98	0.005
	12	Student’s *t*	7	5.02	0.002
	24	Student’s *t*	7	3.29	0.013
	48	Signed Rank	7	11	0.180
Lotilaner (Credelio Quattro)	4	Student’s *t*	7	− 2.21	0.063
	8	Student’s *t*	7	2.09	0.075
	12	Student’s *t*	7	0.34	0.746
	24	Student’s *t*	7	0.24	0.819
	48				NC

NC not calculable all counts equal to zero

## Discussion

These results demonstrate that, compared to untreated dogs, the lotilaner combination product has a faster onset of acaricidal activity against canine *I. scapularis* infestations than the sarolaner and afoxolaner combination products. The rapid onset of lotilaner’s acaricidal efficacy was sustained throughout the 28-day study period. In contrast, acaricidal speed slowed for sarolaner and afoxolaner at 28 days post-treatment compared to the day of treatment (Table 7). While the sarolaner and afoxolaner products performed according to label, there was a clear decline in acaricidal speed for afoxolaner at the 8, 12 and 24 h timepoints on day 28 compared to the equivalent timepoints on day 0. For sarolaner, acaricidal speed was significantly slower at 12 h post-treatment on day 0 compared to 12 h post-infestation on day 28.

A decline in acaricidal speed over the treatment period for the sarolaner combination product against *I. scapularis* was described in two earlier reports. One report described an efficacy of 67.5% and 98.4% against *I. scapularis* at 8 and 12 h post-treatment, respectively, declining to 3.1% and 52.2% at the equivalent times following the day 28 reinfestation [15]. In the other report, sarolaner efficacy against *I. scapularis* decreased from 93.0% at 12 h post-treatment to 27.7% at 12 h post-reinfestation on day 28 [16]. In that study, sarolaner efficacy was 39.0% at 8 h post-treatment and 0.0% at 8 h post-reinfestation on day 28, compared with 49.6% and 8.8% at that timepoint on days 0 and 28, respectively, in the current study. All three of these efficacy assessments used the same source colony of *I. scapularis* for infestations and the same source for study dogs. Possible reasons for the between-study differences may include natural variation in the tick population and individual dogs. Additionally, for investigations of biological systems, between-study variations are inevitable, even when similar or identical protocols are implemented. Nonetheless, the data show a repeatable pattern of declining acaricidal speed of sarolaner (in its combination formulation) against *I. scapularis* during the labeled treatment period. In contrast, lotilaner has shown a consistent sustainment of rapid acaricidal efficacy throughout the labeled treatment period.

The between-study variation in the efficacy of the combination sarolaner product and the decline in its acaricidal speed over the month following treatment has also been demonstrated in studies against the lone star tick, *Amblyomma americanum*. In one study, sarolaner efficacy declined from 97.7% at 24 h post-treatment to 59.3% at 24 h post-infestation on day 28 [17]. At the same timepoints in another study, sarolaner efficacy declined from 74.0% on day 0 to 4.9% on day 28 [18]. The latter study also included afoxolaner, for which efficacy of 97.6% against *A. americanum* at 24 h post-treatment had declined to 0.0% 24 h after reinfestation on day 28. At the 24 h timepoint on day 28 of that study with *A. americanum*, lotilaner efficacy (relative to an untreated control group, 95.3% post-treatment; 92.3% post-reinfestation on day 28) was significantly greater than that of both sarolaner and afoxolaner. Consistent with the current study against *I. scapularis*, there was no significant decline in the acaricidal speed of lotilaner over the study period, while the acaricidal speed of both sarolaner and afoxolaner slowed significantly by day 28.

The accumulated data show that the isoxazolines differ appreciably in their acaricidal speed in dogs. Of the isoxazoline products compared in this study, lotilaner has been shown to have the highest volume of distribution, implying greater adipose tissue partitioning, and the longest half-life (30.7 days) [21–24]. Those pharmacokinetic differences likely account for the sustained higher speed of acaricidal activity of lotilaner, relative to afoxolaner (half-life 15.5 days) [21] and sarolaner (half-life 12 days) [10], throughout the labeled treatment period [10, 21].

The time between tick infestation and pathogen transmission from the tick to the host can vary depending on the tick and pathogen species [25]. The speed of pathogen transmission can be influenced by multiple factors, including the time required to signal pathogen migration to, and replication in the salivary glands, then to enter the host via tick saliva. It is broadly accepted that transmission of *B. burgdorferi* from tick to host requires 24 to 48 h of attachment, and for *A. phagocytophilum,* 16 to 24 h of attachment [1, 26–30]. Therefore, the risk that *I. scapulari*s will transmit pathogens such as *B. burgdorferi* and *A. phagocytophilum* to a canine host increases with the duration of tick attachment. That risk appears to increase exponentially from around 48 h post-attachment, but transmission of these pathogens can occur earlier [31–33]. It has been shown that by quickly killing *I. scapularis,* the isoxazoline class of acaricides can be effective in preventing establishment of *B. burgdorferi* infections in dogs [34–36]. Further studies are needed to determine how well that effectiveness applies to infection with pathogens, such as *A. phagocytophilum*, that can be transmitted more quickly than *B. burgdorferi*. Despite differences in tick-borne pathogen transmission rates, faster acaricidal activity should reduce the risk of tick-borne pathogen transmission.

## Conclusions

Lotilaner has a rapid onset of acaricidal activity that is sustained over the course of a one-month treatment period. In contrast, the onset of acaricidal activity for the afoxolaner and sarolaner endectocidal products evaluated in this study slowed over the treatment period. This difference in acaricidal speed is highlighted by the lower live tick counts in the lotilaner group at early time points immediately post-treatment and post-reinfestation than counts in afoxolaner- and sarolaner-treated groups. Further investigation is needed to determine if the faster acaricidal speed provided by lotilaner translates into reduced risk of canine infection with tick-borne pathogens.

## Supplementary Information


Supplementary material 1.Supplementary material 2.Supplementary material 3.

## Data Availability

Data supporting the main conclusions of this study are included in the manuscript.
