# Supplementary material for: Comparative speed of kill of induced Ixodes scapularis infestations following treatment of dogs with endectocides containing either sarolaner (Simparica Trio™), afoxolaner (NexGard® PLUS), or lotilaner (Credelio Quattro™)
Source: Parasit Vectors. 2026 Jul 17;19:368. doi: 10.1186/s13071-026-07559-y (PMC13523225; doi:10.1186/s13071-026-07559-y)
Supplement: Supplementary file 1 [file 13071_2026_7559_MOESM1_ESM.docx]

**Supplementary table 1.** Summary statistics for untreated and sarolaner (Simparica Trio), afoxolaner (NexGard PLUS) or lotilaner (Credelio Quattro) treated dogs at hours post treatment (Day 0) of existing infestations with 50 *Ixodes scapularis* ticks for each dog placed on Day -2

|  | |  | | | | *Model ln(count+1) (Geometric Mean)* | | | | | | | | | | | | *Model Count Directly (Arithmetic Mean)* | | | | | | | | | | |
| --- | --- | --- | --- | --- | --- | --- | --- | --- | --- | --- | --- | --- | --- | --- | --- | --- | --- | --- | --- | --- | --- | --- | --- | --- | --- | --- | --- | --- |
|  | | |  | | *Compare to Simparica Trio* | | | *Compare to NexGard* | | | *Compare to Credelio* | | |  | | | *Compare to Simparica Trio* | | | *Compare to NexGard* | | | *Compare to Credelio* | | |  |  |  |
| *Tick Count Time* | *Treatment* | | | *Geometric Mean +/- SE^a^* | | | *p-value* | | *Efficacy* | *p-value* | | *Efficacy* | *p-value* | | *Efficacy* | *Arithmetic Mean +/- SE^b^* | | | *p-value* | | *Efficacy* | *p-value* | | *Efficacy* | *p-value* | | *Efficacy* |  |
| 4 hr | Control | | | 25.6+/-2.1 | | | 0.938 | | 6.7% | 0.902 | | -8.5% | 0.904 | | -8.5% | 26.5+/-2.1 | | | 0.845 | | 9.0% | 0.900 | | -7.5% | 0.943 | | -6.1% |  |
|  | Simparica Trio | | | 23.8+/-1.9 | | |  | |  | 0.597 | | -16.3% | 0.600 | | -16.2% | 24.1+/-2.1 | | |  | |  | 0.450 | | -18.1% | 0.525 | | -16.6% |  |
|  | NexGard | | | 27.7+/-2.3 | | |  | |  |  | |  | 1.000 | | 0.1% | 28.5+/-2.1 | | |  | |  |  | |  | 0.999 | | 1.3% |  |
|  | Credelio | | | 27.7+/-2.3 | | |  | |  |  | |  |  | |  | 28.1+/-2.1 | | |  | |  |  | |  |  | |  |  |
| 8 hr | Control | | | 31.9+/-6.2 | | | 0.099 | | 49.6% | 0.110 | | 48.8% | < 0.001 | | 74.5% | 32.1+/-2.7 | | | 0.008 | | 42.8% | 0.005 | | 44.7% | < 0.001 | | 66.9% |  |
|  | Simparica Trio | | | 16.0+/-3.1 | | |  | |  | 1.000 | | -1.6% | 0.125 | | 49.3% | 18.4+/-2.7 | | |  | |  | 0.998 | | 3.4% | 0.200 | | 42.2% |  |
|  | NexGard | | | 16.3+/-3.1 | | |  | |  |  | |  | 0.112 | | 50.1% | 17.8+/-2.7 | | |  | |  |  | |  | 0.262 | | 40.1% |  |
|  | Credelio | | | 8.1+/-1.6 | | |  | |  |  | |  |  | |  | 10.6+/-2.7 | | |  | |  |  | |  |  | |  |  |
| 12 hr | Control | | | 29.3+/-7.7 | | | < 0.001 | | 91.6% | < 0.001 | | 89.5% | < 0.001 | | 89.3% | 30.8+/-2.1 | | | < 0.001 | | 91.1% | < 0.001 | | 82.9% | < 0.001 | | 85.4% |  |
|  | Simparica Trio | | | 2.5+/-0.64 | | |  | |  | 0.969 | | -25.4% | 0.960 | | -28.3% | 2.7+/-2.1 | | |  | |  | 0.839 | | -90.9% | 0.936 | | -63.6% |  |
|  | NexGard | | | 3.1+/-0.81 | | |  | |  |  | |  | 1.000 | | -2.3% | 5.3+/-2.1 | | |  | |  |  | |  | 0.994 | | 14.3% |  |
|  | Credelio | | | 3.1+/-0.83 | | |  | |  |  | |  |  | |  | 4.5+/-2.1 | | |  | |  |  | |  |  | |  |  |
| 24 hr | Control | | | 35.3+/-4.2 | | | < 0.001 | | 99.5% | < 0.001 | | 99.7% | < 0.001 | | 98.8% | 35.5+/-0.8 | | | < 0.001 | | 99.3% | < 0.001 | | 99.6% | < 0.001 | | 98.2% |  |
|  | Simparica Trio | | | 0.2+/-0.02 | | |  | |  | 0.949 | | 52.2% | 0.710 | | -118.9% | 0.2+/-0.8 | | |  | |  | 0.999 | | 50.0% | 0.986 | | -150.0% |  |
|  | NexGard | | | 0.1+/-0.01 | | |  | |  |  | |  | 0.396 | | -357.7% | 0.1+/-0.8 | | |  | |  |  | |  | 0.968 | | -400.0% |  |
|  | Credelio | | | 0.4+/-0.05 | | |  | |  |  | |  |  | |  | 0.6+/-0.8 | | |  | |  |  | |  |  | |  |  |
| 48 hr | Control | | | 35.3+/-2.2 | | | < 0.001 | | 100.0% | < 0.001 | | 99.5% | < 0.001 | | 100.0% | 35.6+/-0.9 | | | < 0.001 | | 100.0% | < 0.001 | | 99.3% | < 0.001 | | 100.0% |  |
|  | Simparica Trio | | | 0.0+/-0.00 | | |  | |  | 0.240 | | NC | 1.000 | | NC | 0.0+/-0.9 | | |  | |  | 0.997 | | NC | 1.000 | | NC |  |
|  | NexGard | | | 0.2+/-0.01 | | |  | |  |  | |  | 0.240 | | 100.0% | 0.2+/-0.9 | | |  | |  |  | |  | 0.997 | | 100.0% |  |
|  | Credelio | | | 0.0+/-0.00 | | |  | |  |  | |  |  | |  | 0.0+/-0.9 | | |  | |  |  | |  |  | |  |  |

NC=Not calculable due to division by zero; ^a^Calculated by the delta method using model based SE;;^b^Model based SE;
