# Supplementary material for: Comparative speed of kill of induced Ixodes scapularis infestations following treatment of dogs with endectocides containing either sarolaner (Simparica Trio™), afoxolaner (NexGard® PLUS), or lotilaner (Credelio Quattro™)
Source: Parasit Vectors. 2026 Jul 17;19:368. doi: 10.1186/s13071-026-07559-y (PMC13523225; doi:10.1186/s13071-026-07559-y)
Supplement: Supplementary file 2 [file 13071_2026_7559_MOESM2_ESM.docx]

**Supplementary table 2:** Summary statistics for untreated and sarolaner (Simparica Trio), afoxolaner (NexGard PLUS) or lotilaner (Credelio Quattro) treated dogs at hours following infestations on Day 21 post-treatment with 50 *Ixodes scapularis* ticks for each dog

|  |  | | |  | | | *Model ln(count+1) (Geometric Mean)* | | | | | | | | | | | | *Model Count Directly (Arithmetic Mean)* | | | | | | | | | | | |
| --- | --- | --- | --- | --- | --- | --- | --- | --- | --- | --- | --- | --- | --- | --- | --- | --- | --- | --- | --- | --- | --- | --- | --- | --- | --- | --- | --- | --- | --- | --- |
|  | | |  | | |  | | | *Compare to Simparica Trio* | | | *Compare to NexGard* | | | *Compare to Credelio* | | |  | | | *Compare to Simparica Trio* | | | *Compare to NexGard* | | | *Compare to Credelio* | | |  |
| *Tick Count Time* | | *Treatment* | | | *Geometric Mean +/- SE^a^* | | | *p-value* | | *Efficacy* | *p-value* | | *Efficacy* | *p-value* | | *Efficacy* | *Arithmetic Mean +/- SE^b^* | | | *p-value* | | *Efficacy* | *p-value* | | *Efficacy* | *p-value* | | *Efficacy* |  |  |
| 4 hr | | Control | | | 30.4+/-2.5 | | | 0.027 | | 25.3% | 0.999 | | -1.1% | 0.007 | | 29.4% | 30.6+/-2.2 | | | 0.046 | | 23.3% | 0.985 | | -2.9% | 0.012 | | 28.2% |  |  |
|  | | Simparica Trio | | | 22.7+/-1.8 | | |  | |  | 0.021 | | -35.3% | 0.932 | | 5.5% | 23.5+/-2.2 | | |  | |  | 0.022 | | -34.0% | 0.932 | | 6.4% |  |  |
|  | | NexGard | | | 30.7+/-2.5 | | |  | |  |  | |  | 0.006 | | 30.2% | 31.5+/-2.2 | | |  | |  |  | |  | 0.006 | | 30.2% |  |  |
|  | | Credelio | | | 21.4+/-1.7 | | |  | |  |  | |  |  | |  | 22.0+/-2.2 | | |  | |  |  | |  |  | |  |  |  |
| 8 hr | | Control | | | 26.9+/-2.6 | | | 0.054 | | 30.1% | 0.849 | | -11.0% | < 0.001 | | 58.6% | 27.8+/-2.0 | | | 0.039 | | 29.3% | 0.807 | | -9.0% | < 0.001 | | 57.7% |  |  |
|  | | Simparica Trio | | | 18.8+/-1.8 | | |  | |  | 0.009 | | -58.8% | 0.004 | | 40.7% | 19.6+/-2.0 | | |  | |  | 0.005 | | -54.1% | 0.047 | | 40.1% |  |  |
|  | | NexGard | | | 29.9+/-2.9 | | |  | |  |  | |  | < 0.001 | | 62.7% | 30.3+/-2.0 | | |  | |  |  | |  | < 0.001 | | 61.2% |  |  |
|  | | Credelio | | | 11.2+/-1.1 | | |  | |  |  | |  |  | |  | 11.8+/-2.0 | | |  | |  |  | |  |  | |  |  |  |
| 12 hr | | Control | | | 23.0+/-4.9 | | | 0.213 | | 47.7% | 1.000 | | -2.6% | < 0.001 | | 86.1% | 23.8+/-2.2 | | | 0.029 | | 40.0% | 0.998 | | -2.1% | < 0.001 | | 78.9% |  |  |
|  | | Simparica Trio | | | 12.0+/-2.6 | | |  | |  | 0.186 | | -96.4% | 0.006 | | 73.4% | 14.3+/-2.2 | | |  | |  | 0.020 | | -70.2% | 0.034 | | 64.9% |  |  |
|  | | NexGard | | | 23.7+/-5.1 | | |  | |  |  | |  | < 0.001 | | 86.4% | 24.3+/-2.2 | | |  | |  |  | |  | < 0.001 | | 79.4% |  |  |
|  | | Credelio | | | 3.2+/-0.69 | | |  | |  |  | |  |  | |  | 5.0+/-2.2 | | |  | |  |  | |  |  | |  |  |  |
| 24 hr | | Control | | | 27.1+/-5.7 | | | < 0.001 | | 97.2% | < 0.001 | | 77.9% | < 0.001 | | 99.7% | 27.3+/-2.0 | | | < 0.001 | | 95.9% | < 0.001 | | 65.6% | < 0.001 | | 99.5% |  |  |
|  | | Simparica Trio | | | 0.8+/-0.16 | | |  | |  | < 0.001 | | -677.9% | 0.378 | | 88.2% | 1.1+/-2.0 | | |  | |  | 0.032 | | -733.3% | 0.983 | | 88.9% |  |  |
|  | | NexGard | | | 6.0+/-1.2 | | |  | |  |  | |  | < 0.001 | | 98.5% | 9.4+/-2.0 | | |  | |  |  | |  | 0.014 | | 98.7% |  |  |
|  | | Credelio | | | 0.1+/-0.02 | | |  | |  |  | |  |  | |  | 0.1+/-2.0 | | |  | |  |  | |  |  | |  |  |  |
| 48 hr | | Control | | | 36.0+/-1.9 | | | < 0.001 | | 100.0% | < 0.001 | | 99.7% | < 0.001 | | 100.0% | 36.4+/-1.0 | | | < 0.001 | | 100.0% | < 0.001 | | 99.7% | < 0.001 | | 100.0% |  |  |
|  | | Simparica Trio | | | 0.0+/-0.00 | | |  | |  | 0.611 | | NC | 1.000 | | NC | 0.0+/-1.0 | | |  | |  | 1.000 | | NC | 1.000 | | NC |  |  |
|  | | NexGard | | | 0.1+/-0.00 | | |  | |  |  | |  | 0.611 | | 100.0% | 0.1+/-1.0 | | |  | |  |  | |  | 1.000 | | 100.0% |  |  |
|  | | Credelio | | | 0.0+/-0.00 | | |  | |  |  | |  |  | |  | 0.0+/-1.0 | | |  | |  |  | |  |  | |  |  |  |

NC=Not calculable due to division by zero; ^a^Calculated by the delta method using model based SE;;^b^Model based SE;
