# Supplementary material for: Comparative speed of kill of induced Ixodes scapularis infestations following treatment of dogs with endectocides containing either sarolaner (Simparica Trio™), afoxolaner (NexGard® PLUS), or lotilaner (Credelio Quattro™)
Source: Parasit Vectors. 2026 Jul 17;19:368. doi: 10.1186/s13071-026-07559-y (PMC13523225; doi:10.1186/s13071-026-07559-y)
Supplement: Supplementary file 3 [file 13071_2026_7559_MOESM3_ESM.docx]

**Supplementary table 3*:*** Summary statistics for untreated and sarolaner (Simparica Trio), afoxolaner (NexGard PLUS) or lotilaner (Credelio Quattro) treated dogs at hours following infestations on Day 28 with 50 *Ixodes scapularis* ticks for each dog

|  | | |  | | | *Model ln(count+1) (Geometric Mean)* | | | | | | | | | | | | *Model Count Directly (Arithmetic Mean)* | | | | | | | | | | |
| --- | --- | --- | --- | --- | --- | --- | --- | --- | --- | --- | --- | --- | --- | --- | --- | --- | --- | --- | --- | --- | --- | --- | --- | --- | --- | --- | --- | --- |
|  | |  | | | *Compare to Simparica Trio* | | | *Compare to NexGard* | | | *Compare to Credelio* | | |  | | | *Compare to Simparica Trio* | | | *Compare to NexGard* | | | *Compare to Credelio* | | |  |  |  |
| *Tick Count Time* | *Treatment* | | | *Geometric Mean +/- SE^a^* | | | *p-value* | | *Efficacy* | *p-value* | | *Efficacy* | *p-value* | | *Efficacy* | *Arithmetic Mean +/- SE^b^* | | | *p-value* | | *Efficacy* | *p-value* | | *Efficacy* | *p-value* | | *Efficacy* |  |
| 4 hr | Control | | | 29.9+/-2.2 | | | 1.000 | | 0.1% | 0.991 | | -3.2% | 0.524 | | 14.1% | 30.5+/-2.4 | | | 1.000 | | 0.8% | 0.982 | | -4.1% | 0.574 | | 14.3% |  |
|  | Simparica Trio | | | 29.8+/-2.2 | | |  | |  | 0.991 | | -3.3% | 0.529 | | 14.0% | 30.3+/-2.4 | | |  | |  | 0.970 | | -5.0% | 0.619 | | 13.6% |  |
|  | NexGard | | | 30.8+/-2.3 | | |  | |  |  | |  | 0.364 | | 16.8% | 31.8+/-2.4 | | |  | |  |  | |  | 0.364 | | 17.7% |  |
|  | Credelio | | | 25.7+/-1.9 | | |  | |  |  | |  |  | |  | 26.1+/-2.4 | | |  | |  |  | |  |  | |  |  |
| 8 hr | Control | | | 23.1+/-2.6 | | | 0.948 | | 8.8% | 0.993 | | -4.6% | 0.004 | | 48.7% | 24.0+/-2.2 | | | 0.864 | | 9.9% | 0.997 | | -2.6% | 0.009 | | 45.8% |  |
|  | Simparica Trio | | | 21.0+/-2.4 | | |  | |  | 0.851 | | -14.6% | 0.015 | | 43.8% | 21.6+/-2.2 | | |  | |  | 0.762 | | -13.9% | 0.047 | | 39.9% |  |
|  | NexGard | | | 24.1+/-2.8 | | |  | |  |  | |  | 0.002 | | 50.9% | 24.6+/-2.2 | | |  | |  |  | |  | 0.005 | | 47.2% |  |
|  | Credelio | | | 11.8+/-1.4 | | |  | |  |  | |  |  | |  | 13.0+/-2.2 | | |  | |  |  | |  |  | |  |  |
| 12 hr | Control | | | 25.0+/-4.5 | | | 0.010 | | 58.0% | 0.963 | | 10.9% | < 0.001 | | 86.8% | 25.5+/-1.7 | | | < 0.001 | | 55.4% | 0.595 | | 10.8% | < 0.001 | | 81.9% |  |
|  | Simparica Trio | | | 10.5+/-1.9 | | |  | |  | 0.028 | | -112.2% | 0.002 | | 68.7% | 11.4+/-1.7 | | |  | |  | < 0.001 | | -100.0% | 0.026 | | 59.3% |  |
|  | NexGard | | | 22.3+/-4.0 | | |  | |  |  | |  | < 0.001 | | 85.2% | 22.8+/-1.7 | | |  | |  |  | |  | < 0.001 | | 79.7% |  |
|  | Credelio | | | 3.3+/-0.59 | | |  | |  |  | |  |  | |  | 4.6+/-1.7 | | |  | |  |  | |  |  | |  |  |
| 24 hr | Control | | | 25.5+/-5.8 | | | < 0.001 | | 100.0% | < 0.001 | | 88.6% | < 0.001 | | 99.6% | 26.3+/-1.6 | | | < 0.001 | | 100.0% | < 0.001 | | 77.6% | < 0.001 | | 99.5% |  |
|  | Simparica Trio | | | 0.0+/-0.00 | | |  | |  | 0.002 | | NC | 0.993 | | NC | 0.0+/-1.6 | | |  | |  | 0.052 | | NC | 1.000 | | NC |  |
|  | NexGard | | | 2.9+/-0.67 | | |  | |  |  | |  | 0.004 | | 96.9% | 5.9+/-1.6 | | |  | |  |  | |  | 0.059 | | 97.9% |  |
|  | Credelio | | | 0.1+/-0.00 | | |  | |  |  | |  |  | |  | 0.1+/-1.6 | | |  | |  |  | |  |  | |  |  |
| 48 hr | Control | | | 29.3+/-3.7 | | | < 0.001 | | 100.0% | < 0.001 | | 99.1% | < 0.001 | | 100.0% | 29.9+/-1.2 | | | < 0.001 | | 100.0% | < 0.001 | | 97.5% | < 0.001 | | 100.0% |  |
|  | Simparica Trio | | | 0.0+/-0.00 | | |  | |  | 0.533 | | NC | 1.000 | | NC | 0.0+/-1.2 | | |  | |  | 0.966 | | NC | 1.000 | | NC |  |
|  | NexGard | | | 0.3+/-0.04 | | |  | |  |  | |  | 0.533 | | 100.0% | 0.8+/-1.2 | | |  | |  |  | |  | 0.966 | | 100.0% |  |
|  | Credelio | | | 0.0+/-0.00 | | |  | |  |  | |  |  | |  | 0.0+/-1.2 | | |  | |  |  | |  |  | |  |  |

NC=Not calculable due to division by zero; ^a^Calculated by the delta method using model based SE;;^b^Model based SE;
